# Reports on Hospitals of the United Kingdom

**Published:** 1914-04-25

**Authors:** Henry Burdett


### REPORTS ON
### Hospitals of the United Kingdom.

By SIR HENRY BURDETT, K.C.B., K.C.V.O.

SERIES III.

### THE ASHFORD COTTAGE HOSPITAL.

This institution was established in 1870, rebuilt in 1878, and enlarged in 1905; and is now vested in the official trustees of Charity Lands under a scheme established by an order of the Charity Commissioners. Six trustees were appointed under the scheme. The hospital is pleasantly situated on a terrace with plenty of garden space at the back, but a good portion of it is not yet cultivated, which seems a pity, seeing that the addition of a well-laid-out garden would add to the attractiveness of the hospital, and at the same time supply the establishment with vegetables and other garden produce.

### The Original Plan Defective.

The original plan was very defective. The lavatories are across the passageways, which separate them from the wards. There is no proper bath accommodation, the two baths provided being in one room off the corridor at the back of the wards. The matron, who has been here for seventeen years, has done her best to work the hospital well, despite the many difficulties due to defective planning. She ought to have most of these defects remedied speedily by the wise action of the trustees. The more recent additions consist of an operation theatre, which faces east. This theatre is difficult to heat owing to its great height and to a large top-light. The latter difficulty might be removed by having a duplicate sheet of plate glass fixed below the top-light, leaving an air-chamber between, which could be heated when necessary and so provide for a current of warm air to pass through it into the theatre. Another addition is the small wing containing the private wards, with separate lavatory accommodation, where paying patients are sometimes received, in addition to those admitted to the pay wards, which seem to have always existed in connection with this hospital.

### Several Good Points.

Good work is said to be done in this hospital by the staff. All kinds of cases are admitted and treated to a conclusion, it being contrary to the practice at this hospital to send the more important cases to larger institutions to be dealt with. Three hundred and seven patients were admitted in 1912, the average mortality being 2.28 per cent.; the average days' residence of each patient was 23.32 days. Anæsthetics were administered 222 times, and 210 operations were performed. The committee in their report state that, "our matron's (Miss Annie Clementson) adeptness as a housekeeper is as noteworthy as her skill in the wards." The matron has to assist her one charge nurse and three probationers, a staff hardly sufficient to work some twenty-five beds and cots, of which twenty on an average are constantly occupied, mostly with severe cases.

### Future Needs.

The hospital at present has no verandahs attached to it, though the wards would be greatly improved by such an addition, and the recovery of the patients might be materially hastened. The pressure on the beds is constantly increasing, and the committee have to look forward to the time when further accommodation will be needed. When that period arrives, we hope they will call in a competent and experienced adviser and so secure that any reconstruction scheme that may be adopted shall include the remodelling of the hospital as it exists to-day, so as to remove most of its structural defects, which naturally add to the cost of upkeep and maintenance. The hospital at present occupies an intermediate position between a modern up-to-date actively administered cottage hospital and a small institution which practically does little real hospital work. The active efficiency of the medical staff and the devotion and competence of an active nursing organisation entitle this hospital to enter the first rank of institutions of its kind, but this promotion must continue to be impossible until the buildings have been reconstructed and remodelled throughout.

### Wanted: an Up-to-date Mortuary.

We were sorry to find, in a hospital which has to provide for the important community the Ashford Hospital serves, that the mortuary was only a large *post-mortem* room whitewashed. It is utterly unworthy of the town of Ashford and the reputation of an institution which has done such excellent service for so many years. We hope some people will interest themselves in this question of the mortuary and provide for its remodelling and reconstruction with the addition of a chapel or view room, which will exhibit proper reverence for the dead and due consideration for the friends of the patients. Once more we must point out that a cottage hospital ought to be a centre of inspiration and a model of efficiency in all matters relating to hygiene and the care of the sick from the commencement to the termination of every illness.

### The National Insurance Act and Voluntary Hospitals.

This hospital has no out-patient department, a fact which is dwelt upon by the committee in their report when dealing with the effects which the Insurance Act may produce directly or indirectly upon all voluntary hospitals. All voluntary hospital managers should keep before them the grave circumstances that at the present time the free admission to voluntary hospitals of insured persons suffering from severe illness involves a costly outlay on the part of these institutions, and that the value of the services rendered represents in the aggregate a large sum in pounds sterling. It follows that were the voluntary hospitals to refuse to admit insured persons to free in-patient benefit, the effect must be financially ruinous to Mr. Lloyd George's scheme of National Insurance, which could not continue solvent without the help of the hospital authorities. The present actuarial basis makes no provision whatever for in-patient benefit for insured persons. The collected payments made to the Commissioners under all heads might, however, be made adequate to provide the cost of such in-patient treatment, as in Germany, were it not for the fact that politics and vote-catching have had so large a share in deciding the principles upon which National Insurance in this country rests at present.

Hospital Managers and National Insurance.

The committee of the Ashford Cottage Hospital deals at length with this question. We reproduce the following statement from the annual report of this hospital: "As to in-patients, our hospital, and not only the hospital, but the patients also, must be very greatly, if not vitally, concerned. The object for which our hospital exists is defined in Rule 1 of our code, as follows: 'The Ashford Cottage Hospital, for in-patients only, is intended for the accommodation of persons residing in Ashford and the neighbouring parishes who, when suffering from sickness or accident, are unable by reason of insufficiency of means to obtain the necessary accommodation, care, and attention; such patients will be admitted free of charge.' It is, therefore, on these conditions that subscribers, collectors, donors, governors, and the medical staff give their money, time, and skill.

"To ensure that the hospital funds are adequate to supply maintenance, nursing, and medicines, as well as treatment for its patients, Rule 17 enacts that 'All patients shall be admitted by subscribers' letters of recommendation, subject to the approval of the House Committee'; and, further, that the hospital may not be imposed upon by persons who are able to pay for the whole or part of such maintenance, nursing, medicines, and treatment, Rule 20 fixes an 'income limit,' and makes provision for 'paying patients,' and 'club patients.' These rules have hitherto been strictly observed, and they have worked satisfactorily.

"But under the National Insurance Act, every one of the patients ordinarily admitted to the hospital under Rule 1 has *some* doctor, who is paid for giving, and is bound to give the necessary treatment in all cases not considered to require unusual or special skill on the part of the medical man; each case has a chemist (or doctor) who is paid to supply, and is bound to supply, the medicines and appliances prescribed for each such insured sick person; and, lastly, each sick insured person is to receive sick pay in addition to the foregoing benefits. Thus it is obvious—in fairness to our subscribers, collectors, donors, and to our own medical staff, all of whom give their money, their time, and their skill to the hospital for the purposes named in Rule 1—that the number of 'insured' persons admissible to our wards, or to the wards of any other hospital, under that rule, ought to be very greatly reduced.

"The Act does, it is true, contemplate that grave cases, dangerous emergencies, major operations, or diseases requiring *special* skill or *special* knowledge from the doctor in attendance, may be and ought to be treated in hospitals; but it does not appear to intend to contribute in any way to the support of the hospitals or the remuneration of the medical man who may spend his time, his skill, or his brains upon such a case.

"It is, therefore, incumbent on all hospital authorities to ascertain concerning every applicant for admission, whether he or she be or be not an 'insured person,' and to lay down rules as to the conditions upon which such 'insured people' may be admitted."

A Point for all Voluntary Hospital Managers.

The committee properly insist that they are the trustees and dispensers of the subscribers' moneys, and that the status of most of the applicants, for whom such instititutions were originally founded, has been profoundly modified by recent legislation. It is essential, therefore, that every voluntary hospital can absolutely rely on the vigilance and assiduity of its officials to see that its treasury is protected against those (insured persons and others) who are not genuine objects for charitable relief at a voluntary hospital.

